# The Associations between Knowledge and Behaviours Related to Touch Screens and Microbiological Threats among IT Students’

**DOI:** 10.3390/ijerph18179269

**Published:** 2021-09-02

**Authors:** Dorota Oszutowska-Mazurek, Jaroslaw Fastowicz, Przemyslaw Mazurek

**Affiliations:** 1 Department of Histology and Embryology, Pomeranian Medical University, Powstancow Wielkopolskich 72 Ave., 70111 Szczecin, Poland; 2 Department of Signal Processing and Multimedia Engineering, West Pomeranian University of Technology Szczecin, 26. Kwietnia 10 St., 71126 Szczecin, Poland; jaroslaw.fastowicz@zut.edu.pl

**Keywords:** user behaviours, microbiological threats, touch screens, data clustering

## Abstract

Current issue like the COVID–19 pandemic show how elementary knowledge and hygiene behaviours are important for ordinary people. Microbiological hazards, not just viruses, can be transmitted in various ways through touch screens. For ordinary users, there is a wide range of behaviours that affect the ability to transfer microbial hazards (viruses, bacteria and fungi). The purpose of the paper is to analyse the association between knowledge and behaviour of touch screen users based on surveys. This paper presents selected results of a survey conducted at the end of 2019 (pre–COVID–19 survey). The survey was conducted on a group of 172 IT school students. The relationship between responses using a 2D linear model regression and clustering is used. Most respondents believe that bacteria were more common than viruses on touch screens. The respondents declare altruism in terms of a greater willingness to lend their smartphone, rather than to use someone else’s. An interesting result is that respondents often lend their smartphone to others, while being aware that viruses or bacteria are present on the touch screens. The results can be used in terms of changes in the education process of smartphone users in relation to microbiological hazards.

## 1. Introduction

The beginning of the 21st century can be characterized as a period of technological optimism. Young people start their life with access to computers, the Internet, mobile devices, and social networks across the world. They use and they trust technology, and technology creates mental stability for users. However, there is a significant difference between using a particular technology and controlling it because of the user’s knowledge and because of external threats. The lack of knowledge, however, gives a false sense of full control, that is very dangerous if unforeseen circumstances arise. The current issue like the COVID–19 pandemic shows how elementary knowledge and hygiene behaviours are important for ordinary people. Some people try to change their behaviour to increase their chances of survival, while others ignore hygiene rules and government recommendations.

Microbiological hazards, not just viruses, can be transmitted in various ways through touch screens. The popularity of touch screens means that they can be an important element in the propagation of microbiological threats. Problems of this type are known and are taken into account to varying degrees by producers and users. For ordinary users, there is a wide range of behaviours that affect the ability to transfer microbial hazards (viruses, bacteria and fungi). It is worth adding that these hazards can have a different character, depending on whether they come from the environment, through a touch screen per person, or between people sharing access to a particular touch screen.

The process of transmitting microbial hazards through touch screens is associated with both touch screen technology, social behaviour of users, hygiene of users, as well as microbiological features of viruses, bacteria, and fungi. The technological aspect and microbiological features are not considered in this paper. There is extensive literature in this field, which is partly presented in Section 1.2.

### 1.1. Human—Machine Interfaces (HMI)

There are two main groups of physical user interfaces:

- Direct contact interfaces (touch based interfaces),
- Touch less interfaces (voice [1,2] and gesture based [3]).

Direct contact HMIs are common methods used in computers, mobile devices, infomats, ATMs (Automated Teller Machines) and many others. Touch leads to bidirectional microbiological contact exchange of viruses, bacteria and fungi between the skin (fingers/hand) and the surface being touched [4]. Mouses and mechanical keyboards are cumbersome to clean and surfaces should be specifically designed for disinfection [5]. The integration of the screen with a touch-sensitive surface is currently the most widely used solution [6]. Properly designed touch screens reduce the number of edges where contamination accumulates. The cleaning of these types of touch screens is simple, which is especially important for touch screens available in public places.

The advantages of touch screens are numerous, including quick cleaning, but it can lead to a false idea that they are not a source of microbiological threats. The problem of accumulation of viruses, bacteria and fungi on the surface of touch screens is known to manufacturers. One of the protection methods is to use special screen coatings [7,8,9]. Another method is to use regular cleaning of touch screens available in public places (infomats, ATMs, ticked and vending machines). The cleaning of personal devices, like smartphones, tablets or smartwatches, is the users’ task, so the microbiological status definitely depends on their behaviour and hygiene aspects [10,11,12,13].

Some screens have a coating that reduces the risk of viruses, bacteria and fungi [14], but the actual effectiveness is the subject of research. In addition, not all touch screens have any protection against microbiological threats. Regardless of the technical aspects of touch screens, behaviour and hygiene of users have a significant impact. The assessment of these aspects is important for determining potential sources of issues and the need for education.

### 1.2. Related Works

#### 1.2.1. Medical Staff and Mobile Devices

It is worth noting that many studies mainly concern health professionals or students of health sciences, and detailed information is written in Supplementary [11,13,15,16,17,18,19,20,21,22,23,24,25,26,27,28].

#### 1.2.2. Ordinary Users and Mobile Devices

According to [29] cellular phones were contaminated with *Micrococcus* and *Staphylococcus* species.

It was also described that [30] *Candida* species colonised mobile phones and hands of users; 74.9% cellular phones were colonised. Predominant species were isolated: *Candida glabrata*, *Candida albicans* and *Candida krusei* isolated. The survey revealed that only 19.4% users cleaned the surface of cellular phones.

According to [31] 97.9% touch screen mobile phones (TMP) and 98.3% keypads devices (KMP) were contaminated with microorganisms. It was observed that significant difference was connected with the screen size of devices. The most predominant microorganisms present were *Staphylococci*, *Enterococcus/Sterptococcus* and *Escherichia coli*. There was no significant difference between TMP and KMP according to bacterial contamination, but touch screen devices had significantly higher microbial load than keypads devices (p<0.05). A total of 205 cell phones/devices were tested for microbial contamination: 76 devices were owned by healthcare professionals and 129 devices belonged to the non—healthcare professionals group. There were no significant differences detected at the rate of microbial contamination.

Isolated and identified bacteria from covered and uncovered mobile phones are considered in the work [14]. Devices harboured the following microorganisms: *Bacillus* species, *Staphylococcus aureus*, *Staphylococcus epidermidis*, *Escherichia coli*, *Pseudomonas* species and *Serratia* species. After treatment with 95% of ethanol and covering with plastic covers for one week, the identification of microorganism was performed and revealed that the microorganisms’ growth was reduced to 90%.

An interesting study was described in [10]. The growth of microorganisms was checked in samples from mobile phones and hands of parents of neonatal infants before and after applying of anti–microbial gel. All devices demonstrated microbiological contamination; 22% demonstrated no growth on hands after treatment with antimicrobial gel, and only 38% of parents admitted cleaning mobiles at least weekly.

There was also a paper [32], that indicated that cellular phones may indicate personal microbiome of the owners −22% of bacteria isolated from fingers were present on touchscreen smartphones.

It was described in [33] that hand hygiene plays an essential role in preventing from infections and spreading pathogens; 92.9% of cellular phones were contaminated with bacteria and it was observed that bacterial growth was detected in 18.6% plaques inoculated with disinfected fingers.

Swab samples were collected from four groups in the study [34]: food vendors, lecturers/students, public servants and health workers. Devices from food vendors harboured the highest rate of contamination. *Coagulase Negative Staphylococcus* was the most prevalent in mobile phones belonging to food vendors, and least in the samples collected from devices of health care workers. Other identified bacteria were *Enterococcus faecaalis*, *Pseudomonas aeruginosa*, *Escherichia coli* and *Klebsiella* species.

The study [35] showed that the colonisation of smartphones was limited to human skin microorganisms.

It was depicted in study [36], that keypad mobile phones are more contaminated than touch screen devices and are more likely to harbour pathogenic bacteria. Out of all 316 contaminated cell phones, the highest contamination was observed in hospital doctors and staff (100%), and then medical students (92%), lecturers and university employees (87%) and the lowest number among people not related to the health service (45%); 45% is a high percentage of contaminated mobile phones. Therefore, it is significant from epidemiological point–of–view and should be taken into consideration.

### 1.3. Contribution and Content of the Paper

The aim of the study was to investigate the relationship between the knowledge and behaviour of touchscreen users, in particular smartphones, based on anonymous questionnaires addressed to computer science students. The questionnaires were intentionally directed to the group of respondents who were not representatives of the health service. Moreover, the young age of the respondents highlights the future possibility of earlier modelling of attitudes related to the proper use of touchscreen devices, especially in the context of the COVID–19 pandemic.

Based on the analysis of the literature, a large number of works related to the subject are available in the group of health care workers and medical students. Meanwhile, the problem of transmitting microbiological threats is a more general problem that affects all the people, especially young who sometimes use smartphones for hours. As the research was carried out before the COVID–19 pandemic, it presents the behaviour of touchscreen users during and in the early stage of the pandemic development (early 2020) and is an important source of information on transmission methods. In the later period of the pandemic, there were changes in the behaviour of users, due to formal restrictions. The obtained results are important in further monitoring of changes in userbehaviour over a longer period of time (this requires the repetition of the survey).

- Research on subjective beliefs of the presence of viruses, bacteria and fungi on touch screens are modelled.
- Research on the ability to share a smartphone with others with signs of illness or lack of personal hygiene is presented. Clustering and linear models were used for the analysis.
- Subjective associations between microbiological threats for personal contact situations using linear models are modelled.

## 2. Materials and Methods

This paper presents selected results of a survey conducted at the end of 2019 (pre–COVID–19 survey). The survey was conducted on a group of 172 IT school students. The age of the students/pupils is between 13–18 years old with Mean (SD): 15.92 (0.93). The survey covered 165 questions about hygiene and knowledge and the use of different touch devices. In the 2020/2021 school year, there were 1,899,321 secondary education students in Poland and 647,145 students in technical schools were studying in this country. In the West Pomeranian Voivodeship, 25,689 students attended technical secondary schools [37]. The survey involved all students of 1st and 2nd year in one technical secondary school (172 students compared to 336 all students of a technical secondary school). This technical secondary school is private; however, education is free for all students. The technical secondary school has a very high ranking in the voivodeship. Most of the students are fromthe city of Szczecin (the capital and largest city of the West Pomeranian Voivodeship in northwestern Poland) and the neighbouring cities/region.

At the beginning of the survey, people were informed about the purpose of the survey. The survey was computer–based (using Moodle) and could be completed at any time. Completing the survey was unidirectional and the respondent could not change earlier answers. The survey was anonymous and began with questions about personal data such as age or gender. The next parts concerned used touch devices, hygiene related to touch screens, behaviours related to touch screens and personal hygiene. At the end of the survey there are questions about the subjective assessment of bacteria/viruses/fungi on touch screens.

Some surveys were very incomplete because the respondent would stopped filling it after a few questions, so they were rejected. The final number of surveys completed in full or within the scope of questions considered in this paper is N=172. Since some questions were conditioned by previous answers, there are data gaps that have been supplemented using imputations. The Mice algorithm was used for imputation [38,39]. There are 162 fully completed questionnaires in the scope of the questions considered in the paper. The R software (www.r-project.org, accessed on 7 June 2021) v.3.6.3 was used to analyse the data.

The questions were formulated using continuous scale answers. For technical reasons, a series was used, typically 11 radio buttons placed horizontally with a textual description of the border responses (Figure S1 in Supplementary Materials). This means that there were no binary or trinary answers for most questions. This is a deliberate approach because most of the questions are subjective.

The questions from the subset of the full survey considered in this paper are presented in the Table 1 and Figure S1.

Boundary values assigned to labels are: ‘never’ (−1.0), ‘it happened often’ (+1.0); ‘never’ (−1.0), ‘it’s not a problem’ (+1.0); ‘none’ (0.0), ‘a lot of them’ (+1.0). The market price range of currently owned smartphone was selected based on market analysis. Smartphones above PLN 2,100 were grouped together (PLN is Polish zloty currency symbol, in 2020: 1 PLN ∼ 0.22 €).

During the development of the questionnaire, its validation was carried out. The questionnaire was developed after several iterations, with the participation of several people who completed and indicated incompleteness, redundancy, and ambiguity of some questions.

For the questions considered in this publication, an internal consistency analysis was performed using Cronbach’s alpha. The value of this coefficient is 0.999 which results from the large number of responses in relation to the number of questions and the existence of correlation between the questions. Correlation between questions is expected as they concern a narrow range of similar actions.

This paper shows analyses the relationship between responses using a 2D linear model regression. In addition, grouping of responses is considered and the analysis uses PCA (Principal Component Analysis) [40]. Two clustering approaches, using k–means and k–medians are applied.

## 3. Results

### 3.1. Subjective Associations between Expected Microbiological Threats

There are three independent questions about the belief that bacteria, viruses and fungi are present on touch screens. The association between the answers shows the relationship and the dominance of beliefs.

**Figure 1:**
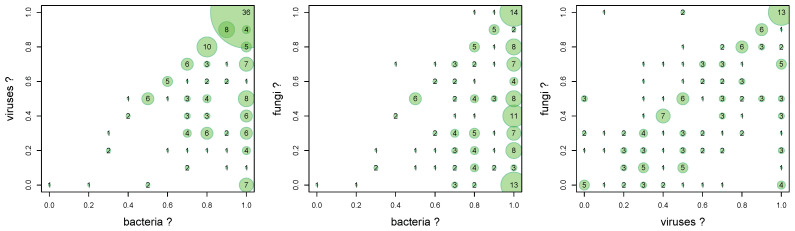
Comparative 2D histogram (“Do you think there are bacteria/viruses/fungi on touch screens?”), 0.0—none, 1.0—a lot of them.

A comparative 2D histogram is shown in Figure 1 with number of particular cases.

The Goodman–Kruskal’s gamma test [41] for association analysis is applied. The results are show in the Table 2.

Gamma values show (Table 2) that users have a moderate confidence in the simultaneous presence of bacteria and viruses (0.39) and viruses and fungi (0.48) on touch screens.

The belief in the simultaneous presence of bacteria and viruses is relatively weak (0.18). This is also seen in the graph (Figure 1 middle) where the fungi axis does not significantly affect the 2D distribution. Additionally, this chart shows that the belief in the presence of bacteria is very strong. Most responses are concentrated at bacteria=1.0, regardless of the fungi axis.

The association graph between viruses and bacteria (Figure 1 left) shows that virtually all interviewees believe that bacteria are more likely than viruses on touch screens. This is shown as a blank top triangular area in the graph. Much of the answer is in the area of the strong belief that viruses and bacteria are present simultaneously. This means that there is little awareness of the presence of viruses, or viruses are treated as a marginal threat.

This is similar to the association between fungi and bacteria (Figure 1) where there is also an almost empty upper triangular area in the graph.

### 3.2. Subjective Associations between Expected Microbiological Threats and Market Price of Smartphone

**Figure 2:**
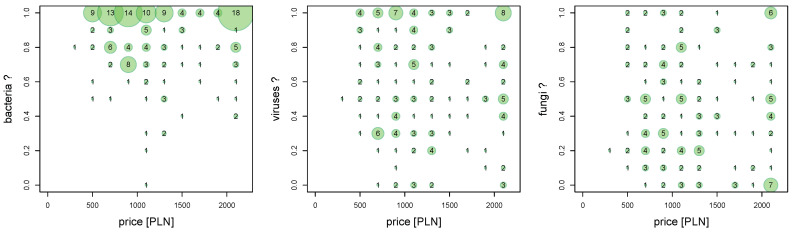
Associations “Do you think there are bacteria/viruses/fungi on touch screens?” and smartphone market price (prices above 2100 PLN are saturated).

This study shows whether there is an association between the price of a personal smartphone and the occurrence belief of biological threats on touch screens (Figure 2). It is worth noting that the most commonly used touch screen for students of this age is the screen of a personal smartphone. No questions were asked about the biological threats associated withsmartphone owned.

The Goodman–Kruskal’s gamma test [41] for association analysis is applied. The results are shown in the Table 3.

More expensive smartphone models usually have better screen protection against scratching, cracking due to impact, as well as protective coatings that reduce microbiological hazards.

There is no relationship between the price of the smartphone owned by the respondent and the belief that there are microbiological threats. Gamma values are close to zero (Table 3).

The lack of such a relationship is interesting because there is no relationship between awareness of specific microbiological hazards and the status of having a cheap or expensive smartphone. It should be noted that the survey did not ask whether the purchase of a specific smartphone was related to the protection against microbiological threats.

### 3.3. Subjective Associations between of Social Behaviours Related to Giving Own Smartphone to Other Persons and Using Someone Else’s Smartphone

**Figure 3:**
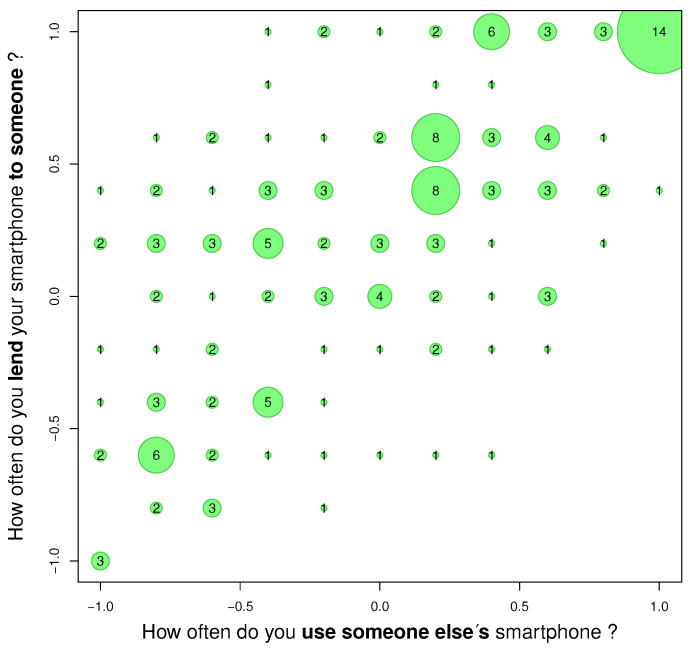
Associations “How often do you use someone else’s smartphone?” and “How often do you lend your smartphone to someone?”.

Another association concerns the subjective lending of own smartphone to other people in relation to using someone else’s smartphone (Figure 3. This relationship is partly related to the user’s altruism, but lending own smartphone may be associated with the desire to show the collected data, for example photos, or even the desire to show off.

The results of Goodman–Kruskal’s gamma test are shown in the Table 4.

The Figure 3 can be a measure of users’ altruism. It shows a greater willingness to lend their smartphone to other people than to borrow. This is seen as an upward shift in the answer. The gamma value is 0.57 (Table 4).

From the point of view of transmission, this means that the studied group unknowingly supports the transmission of microbiological hazards in general with their behaviour. The smartphone is not a fully personal device. Hence, the next analysis examined the relationship between potential social barriers to transmission.

The transmission is not only unidirectional but can be bidirectional. As smartphones are stored in pockets, a pocket itself is an additional transmission element. Microbiological analysis in this regard is worth attention.

### 3.4. K–Means and K–Median Clustering of Social Behaviours Related to Giving Own Smartphone to Other Persons

**Figure 4:**
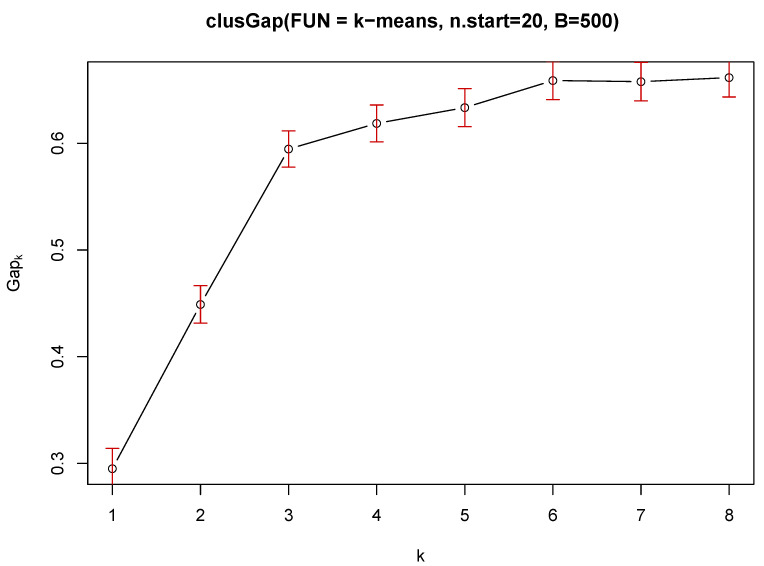
The gap statistics (k—means, Euclidean distance).

This study is about social behaviours and the grouping of them. Two types of clustering were used to assess the common characteristics of users. The main problem in the clustering task isan estimation of the number ‘k’ of clusters [42]. The gap statistic for estimating the number of clusters was used for the k–means method and Euclidean distance: R:clust(2.1.0):clusGap:Tibs2001SEmax [43] suggests k=3 and R:clust(2.1.0):clusGap:firstSEmax suggests k=4. The separation between clusters is not perfect, which is typical, that is why the gap statistic curve increases, although its saturation occurs (Figure 4). The number of clusters has been limited to 8.

Two cases k=3 and k=4 are analyzed using R:flexclust:kcca (k–centroid cluster analysis) [44]. This function also allows the clustering using Manhattan distance for k–medians estimator. The Manhattan metric is important because questions are independent, and the Euclidean metric assume spatial dependence between them.

**Figure 5:**
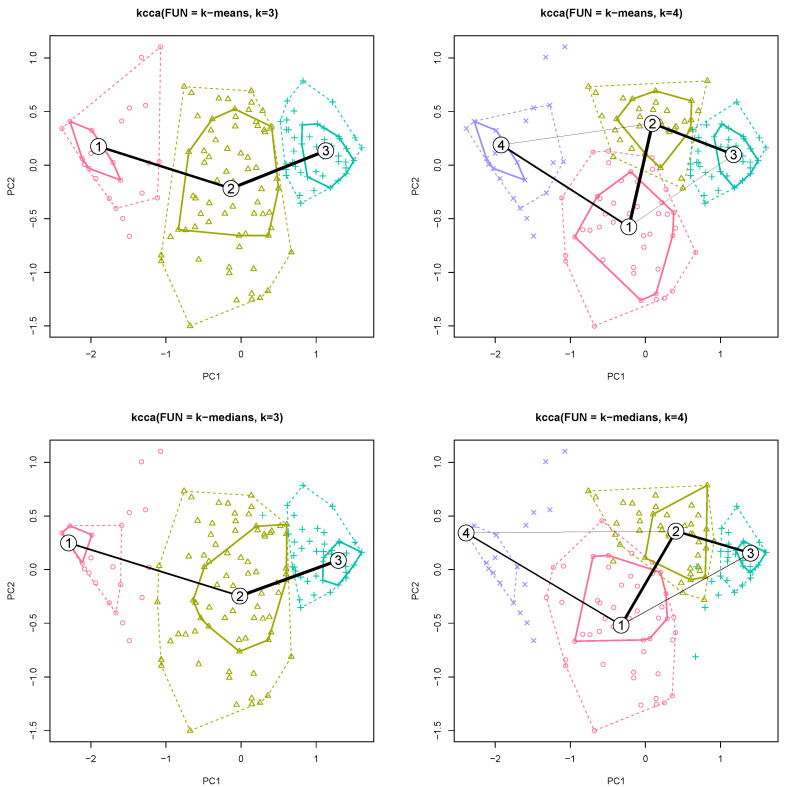
PCA plot for k—means and k—medians; *k* = 3 and *k* = 4 (solid line encloses 50% and dotted line encloses 95% of respondents in clusters).

A graphic representation of clustering is shown in Figure 5 for two first components (PC1, PC2). The location of the centroids in relation to the survey questions is presented in the Tables S2 and S3. This table allows to specify the average answer value for a given question for a given cluster.

To investigate the social barriers to transmission by lending own smartphone to other people, clustering was used for two centroid methods (mean and median) for a different number of groups (*k*). The number of groups was selected (k=3) based on a gap statistic analysis (Figure 4).

Increasing the number of clusters makes it possible to clarify the division of groups (the values are prioritized in Tables S2 and S3). The additional cluster for k=4, however, does not affect the changes in the centroids obtained for k=3, but only the division of the centroid (no. 2) k=3 into two more (no. 1 and 2) is visible in the figure (Figure 5, k=4). The clustering result is similar for both criteria (mean and median).

For k=3, clusters no. 1 and no. 3 (corresponding to clusters no. 4 and no. 3 with k=4) are extreme. Cluster no.1 is a group that will lend their smartphone to a visibly sweating person (0.800), can lend their smartphone to a visibly dirty person (0.554), see no problem with a runny nose (0.880) and visible skin changes (0.760). This group is unknowingly positively transmission-oriented.

Cluster no. 3 is a group that is negative about lending a smartphone: will not lend it a visibly sweating person (−0.725), will not lend smartphone to a visibly dirty person (−0.482), see problem with a runny nose (−0.668) and visible skin changes (−0.823). This group limits the transmission of microbiological hazards.

Cluster no. 2 is the middle group that will be reluctant to lend a smartphone to a: visibly sweating person (−0.200), visibly dirty person (−0.482) and with visible skin changes (−0.225). In case of problem with a runny nose the value is quite high (0.345). This is the group for which there is social acceptance of the runny nose problems.

After increasing the number of groups to k=4, the intermediate group (no. 2, k=3) is divided into two with higher polarization for almost all cases. The exception is the visibly dirty person criterion: both new groups do not accept visibly dirty people.

### 3.5. Subjective Associations between Expected Microbiological Threats and Behaviour Using 2D Linear Model

In addition, a linear relationship (R:stats:lm(3.6.3)) between belief in the amount of bacteria/viruses/fungi on touch screens and the price of the user’s smartphone was examined. Results are shown in Tables S4–S6.

In the association analysis, a linear 2D model with three parameters was adopted. Two of them are independent beliefs of the interviewee, and the third is the intercept (constant). The result is an absolute frequency of responses for three independent classes (presence of bacteria/viruses/fungi on smartphone screens). The absolute frequency of the answers can be changed into a probability because the number of all answers is known (N=172).

The frequency of responses that create an association between the price of a smartphone and the presence of microbiological hazards on touch screens is not linearly modelled (Table S4 top).

The linear model of the response rate is for the association of lending a smartphone to another person and the belief in the presence of microbiological hazards on the touch screen. Since the personal smartphone belongs to the general group of touchscreens, it means that users treat microbiological hazards on the screens as negligible or they treat their smartphone as not transmitting threats (Table S4 middle).

The linear model is also associated with lending a phone to people clearly sweating and the belief about the presence of fungi on touch screens (Table S4 bottom), but the sign of the coefficient is negative. This means that as the belief in the presence of fungi grows, the desire to lend a smartphone to people who are sweating decreases. A similar association, but for all types of microbiological hazards, is for people lending a smartphone to clearly dirty people and people with visible skin changes, where the coefficients are significant and negative (Table S5 1st row and 3rd row respectively).

There is no association in the linear model for person coughing or with a runny nose and their opinion about microbiological hazards. This can be interpreted as the social acceptance of this group of people.

There is no linear association between using someone else’s smartphone and and their opinion about microbiological hazards (Table S5 4th row). A smartphone that is borrowed from another person may be dirty or carry sweat marks. In the case of dirt, there is no association with the opinion on microbiological hazards on touch screens (Table S6 top).

In the case of sweat marks (Table S6 bottom), there is an association with the opinion on microbiological hazards on touchscreens for bacteria and viruses. The value of the coefficient for the belief about the presence of bacteria is relatively high, over 9, and the willingness to use such a smartphone israther limited because the coefficient is negative below −2. The intercept factor is additionally negative below −4. This means that the respondents associate the presence of sweat with the transmission of threats by bacteria. A linear relationship, but much weaker, is for the presence of viruses. The belief in the presence of viruses has a factor of almost 1, and the desire to use such a smartphone is almost −1.

### 3.6. Subjective Associations between Microbiological Threats for Personal Contact Situations Using 2D Linear Model

The model (R:stats:lm(3.6.3)) for absolute frequency is shown in Table S7. This model assumes a 2D histogram and a plane fit (freq∼x+y) in 3D space using a 2D regression, where freq is the frequency of the specified x,y pair cases.

Association analysis with a 2D linear model for lending a smartphone to other people and using someone else’s without taking into account the opinion on microbiological hazards is presented in Table S7.

In all cases, the intercept value is positive and significant in the range of 2–2.5. The 2D linear model is forlending your smartphone to dirty people (opinion rather negative because the coefficient is −1.105), to people with clearly visible skin changes (opinion rather negative because the coefficient is −0.7552). The desire to use someone else’s dirty smartphone is also negative as the coefficient is −1.1551.

### 3.7. Responses for 50% Threshold Level Value

Since all the studies considered in Section 1.2 use binary indicators, the 50% threshold was used for discussion in order to obtain results with the same data type (Table 5).

## 4. Discussion

The study was based on a questionnaire; it was not possible to conduct microbiological tests of students’ smartphones, therefore the study was behavioural. The study was conducted prior to the COVID–19 pandemic. Therefore, there is a different view of the results from the current perspective [45,46] and answers of respondents from our study might be different due to the pandemic. It can be associated with a probable change in the behaviour of people, both health professionals and other groups. In the available publications [10,11,12,15,19,30,47,48,49,50,51] the respondents were mainly adult healthcare professionals and students of health sciences. It is valuable to gain knowledge of the behaviour associated with the use of touchscreen devices, including smartphones, in groups not related to health care, which may be of particular importance, especially in the current context of the COVID–19 pandemic. In [10] parents of neonates were examined. In our study, the respondents were young individuals, students of IT technical secondary school, which is already a novelty in the study. Moreover, the questionnaires from the available literature are based on questions with specific answer variants, e.g. binary—yes/no choice. Clustering has not been performed in these studies. Clustering is a source of valuable knowledge that is acquired automatically without a priori assumptions or excessive model simplifications forced by simplified survey responses.

Subjective responses should be treated in the context of Bayesian statistics based on distributions. To obtain correct distributions, it is necessary to use more responses from the continuous range. For practical reasons, discrete distributions were used [52]. Although this paper did not use the Bayesian analysis methods directly, it was possible to use clustering and two–dimensional regression analysis. The obtained distributions show that while some dependencies can be modelled with linear distributions, others cannot. This means that the adopted approach is different comparing approaches used by other authors where the answers to the survey questions are binary or have three or four options [11,12,15,23,30,47,48,49,51]. The essence of the work is that the entire questionnaire was prepared in such a way that practically all the questions allow obtaining distributions for almost all responses.

For practical reasons, the objectification of subjective data with the use of de Finetti’s game [53] was unfortunately rejected, because answering each question would require a few further subquestions for each question, which would make it difficult to obtain answers from respondents due to excessive time necessary and their weariness.

In further works, it is planned to use the methods of automatic data mining to automatically create models describing the associations between the data from the questionnaire, both for the obtained distributions and for individual survey results.

The applied approach, which is based on the analysis of distributions, rejects the use of statistical methods based on typical estimators, such as mean, standard deviation, because the distributions are not unimodal.

In this work, associations between pairs of distributions were analysed, the pairs selected arbitrarily.

The essence of the publication is the analysis of the association between the declared behaviours of users and their conviction about the presence of microbiological hazards on touch screens, the focus being on touch screens of smartphones, but the survey also covers other devices with touch screens.

The survey was conducted in one school for organizational reasons due to the need to maintain the anonymity of respondents under the control of the school’s IT staff using the internal Moodle server. The use of the internal Moodle server made it possible to block multiple completion of the survey by one student and at the same time made it possible to fill in parts of the survey in several sessions.

The ability to complete the questionnaire in several sessions required the student’s identification, but only within the school’s internal IT infrastructure; therefore, the participation of IT administrators was necessary. Repeating this type of procedure in other schools would be troublesome.

The questionnaire contains questions on hygiene, but the subject of this article is not to analyse behaviour related to hygiene, which will be the subject of further analysis and subsequent publications.

Interesting results were obtained on the awareness of microbial contamination of touchscreens compared with other authors’ research in the medical community, which focused only on smartphones.

In our study, respondents had a moderate belief about the presence of bacteria, viruses and fungi on touch screens at the same time. Virtually 92% of respondents believed that bacteria are present in greater numbers than viruses on touch screens. There was a moderate awareness of the presence of viruses on devices in 60% of respondents; 42% of respondents were convinced about the presence of fungi on touchscreens (Table 5). In [10], 92% of parents of newborns believed that there could be germs on their phones, the question was about germs in general, without being divided into bacteria, viruses and fungi.

In [48] most of the respondents studied microbiology and they were largely convinced that microbes could grow on phones.

In publication on the of paediatric hospital staff [49] 98% of respondents believed that telephones could be microbiologically contaminated.

In [30] 81% of respondents believed that phone surfaces could be inhabited by microorganisms, including bacteria (80% of respondents), fungi (60% respectively) and viruses (28.6%); 10.2% of the respondents had difficulties answering the question.

Some publications have considered lending the phone to others as a potential source of increased microbial contamination [23,48]. In [23] 95.5% of healthcare workers said they shared the phone with colleagues at work and at home.

An interesting study concerned computer keyboard sharing, which indicated the importance of taking into account sharing of other electronic devices, including smartphones [4] and a fact that contamination of devices depended largely on the behaviour of users, not only related to sharing equipment with other users.

Our study obtained an interesting result, as it turned out that 68% of respondents are likely to lend a smartphone (68%) to another person. Subjective questions about sharing phones with other users were also included in relation to possible symptoms related to the health condition, e.g. a person with visible skin lesions, with a runny nose or coughing. These additional data were not included in other research in this field.

## 5. Conclusions and Further Work

Most respondents believed that bacteria were more common than viruses on touch screens. This may be due more to education on microbiological hazards, where more attention is paid to bacteria than viruses.

There are no connections in terms of beliefs regarding microbiological hazards on touch screens, and the fact that less wealthy people may have more expensive smartphones. Although more expensive smartphones may have better microbiological protection in terms of protective coatings, this is not an important element that users pay attention to.

The respondents declare altruism in terms of a greater willingness to lend their smartphone than to use someone else’s. Three groups can be distinguished: positive, negative and reluctant in terms of offering a smartphone to other people potentially transmitting microbiological hazards. While extreme groups are highly polarized (positive and negative), the reluctant group shows that runny nose problems are socially acceptable. This may indicate that the transmission of microbial hazards is easier then, as this group joins the transmission support group.

The paper shows various relationships with the use of linear models for all respondents. An interesting result is that respondents often lend their smartphone to others, while being aware that viruses or bacteria are on the touch screens (non–direct question, Table S4 2nd row). This shows that the knowledge about the presence of bacteria and viruses and the awareness of the distribution of threats are not properly related to each other.

The results can be used in terms of changes in the education process of smartphone users in relation to microbiological hazards.

The advent of COVID–19 has forced changes in people’s hygiene. For this reason, it is planned to continue the research and compare the results with the current behaviour. In 2020 there were very limited contacts of students at school due to remote learning, therefore the survey was not repeated. In 2021 the restrictions are being gradually lifted and remote learning at school is being abandoned. We plan to repeat the survey in the autumn and winter of 2021.

The question is how much COVID–19 has affected changes in hygiene behaviour. The transmission of microbiological hazards through touch screens for example of smartphones, as shown by the work of many research teams, is a real issue (Section 1.2). The main question is to what extent the change in behaviour (2020) towards greater hygiene will be permanent among the studied group in the following years (2021 and beyond). A failure to maintain hygiene by disregarding the problem may constitute information about potential problems of COVID–19 recurrence and other threats. 

## Figures and Tables

**Table 1:** Questions considered in this paper.

Question	Range and Number of Responses
Smartphone market price	(300) ⋯ (>2100) PLN
	162 (91.5%)
Did youlend your smartphone	never⟺it happened often
to another person?	167 (94.3%)
Do you ever use someone else’s	never⟺it’s not a problem
smartphone?	167 (94.3%)
Would you use someone else’s	never⟺it’s not a problem
smartphone that is dirty?	166 (93.8%)
Would you use someone else’s	never⟺it’s not a problem
smartphone that is wet with sweat?	167 (94.3%)
Would you lend your smartphone	never⟺it happened often
to a person who is visibly sweating?	167 (94.3%)
Would you lend your smartphone	never⟺it happened often
to a visibly dirty person?	167 (94.3%)
Would you lend your smartphone	never⟺it happened often
to a person coughing or with a runny nose?	167 (94.3%)
Would you lend your smartphone	never⟺it happened often
to a person with visible skin changes?	165 (93.2%)
Do you think there are bacteria on touch screens?	none⟺a lot of them
	167 (94.3%)
Do you think there are viruses on touch screens?	none⟺a lot of them
	166 (93.8%)
Do you think there are fungi on touch screens?	none⟺a lot of them
	166 (93.8%)

**Table 2:** Goodman–Kruskal’s gamma statistic for subjective associations between expected microbiological threats.

Pair Type	Goodman—Kruskal’s Gamma	CI 95%	*p*-Value
bacteria—viruses	0.39	0.27, 0.52	$1.2\times 10^{-8}$
bacteria—fungi	0.18	0.04, 0.31	0.01
viruses—fungi	0.48	0.37, 0.59	$6.7\times 10^{-16}$

**Table 3:** Goodman–Kruskal’s gamma statistic for subjective associations between expected microbiological threats and market price of smartphone.

Pair Type	Goodman—Kruskal’s Gamma	CI 95%	*p*-Value
price–bacteria	−0.03	−0.18, 0.11	0.66
price–viruses	−0.06	−0.18, 0.07	0.37
price–fungi	−0.06	−0.19, 0.07	0.34

**Table 4:** Goodman–Kruskal’s gamma statistic for subjective associations between social behaviors related to lending own smartphone to other persons and using someone else’s smartphone.

Pair Type	Goodman—Kruskal’s Gamma	CI 95%	*p*-Value
using not own—giving own	0.57	0.47, 0.66	$2.2\times 10^{-16}$

**Table 5:** Responses for 50% threshold.

Question	Responses
Did you lend your smartphone	68%
to another person?	
Do you ever use someone else’s	50%
smartphone?	
Would you use someone else’s	49%
smartphone that is dirty?	
Would you use someone else’s	22%
smartphone that is wet with sweat?	
Would you lend your smartphone	36%
to a person who is visibly sweating?	
Would you lend your smartphone	22%
to a visibly dirty person?	
Would you lend your smartphone	51%
to a person coughing or with a runny nose?	
Would you lend your smartphone	33%
to a person with visible skin changes?	
Do you think there are bacteria on touch screens?	93%
Do you think there are viruses on touch screens?	60%
Do you think there are fungi on touch screens?	42%

## Supplementary Materials

The following are available at https://www.mdpi.com/article/10.3390/ijerph18179269/s1, Figure S1. Fragment of the questionnaire with the questions considered in the article (questionnaire in Polish). Table S1. Questions considered in this paper. Table S2. Centroid locations for k–means. Table S3. Centroid locations for k–medians. Table S4. Absolute frequency for 2D linear model of associations (N = 172) (part 1/3). Table S5. Absolute frequency for 2D linear model of associations (N = 172) (part 2/3). Table S6. Absolute frequency for 2D linear model of associations (N = 172) (part 3/3). Table S7. Absolute frequency for 2D linear model of associations (N = 172).

## Abbreviations

The following abbreviations are used in this manuscript:

ATM	Automated Teller Machine
CI	Confidence Interval
CNS	*Coagulase Negative Staphylococcus*
COVID	COronaVIrus Disease
DNA	DeoxyriboNucleic Acid
HMI	Human–Machine Interface
IR	Infrared Light
IT	Information Technology
MCD	Mobile Cellular Device
MRSA	*Methicillin Resistant Staphylococcus Aureus*
KMP	Keypads Device
PC	Principal Component
PCA	Principal Component Analysis
PLN	Polish zloty (currency)
SD	Standard Deviation
TMP	Touch screen Mobile Phone
