# Supplementary material for: The Associations between Knowledge and Behaviours Related to Touch Screens and Microbiological Threats among IT Students’
Source: Int J Environ Res Public Health. 2021 Sep 2;18(17):9269. doi: 10.3390/ijerph18179269 (PMC8431698; doi:10.3390/ijerph18179269)
Supplement: Supplementary file 1 [file ijerph-18-09269-s001.zip › ijerph-1272967-supp-edited.pdf]

Supplementary Materials: The Associations Between Knowledge and Behaviours Related to Touch Screens and Microbiological Threats Among IT Students'

Dorota Oszutowska–Mazurek [1,†,*], Jaroslaw Fastowicz [2,†] and Przemyslaw Mazurek [3,†,*]

1. Medical Staff and Mobile Devices

There is a lot of research regarding mobile devices of medical staff and bacterial contamination and the survey is described in [1]. This work shows that recent studies found that between 9% and 15% of MCDs (Mobile Cellular Devices) carry pathogenic bacteria. Recommendations are to practice good hand hygiene, restrict MCDs in high–risk areas, and sanitize MCDs devices using 70% isopropyl alcohol to reduce contamination.

In study [2] revealed that one fifth of mobile phones harboured pathogenic microorganisms

Coagulase Negative Staphylococcus (*CNS*) was the most frequently isolated microorganism from cellphones belonging to doctors and paramedical staff in departments at government medical college and hospital, Amritsar [3]. Coagulase negative staphylococci are normal flora of the skin so inevitable contamination occurs. These can cause device related infections but are ubiquitous in the hospital environment. Hence, contamination is likely a reflection of use by the same person rather than hand hygiene. The results indicated that 40% of devices were contaminated and the decontamination with 70% isopropyl alcohol was 98% effective.

Mobile phones of health care workers also harboured microorganism according to [4]. In samples from mobile phones of resident doctors *Coagulase Negative Staphylococci* (71.87%) *Diphtheroids* (21.87%), *Aspergillus niger* (6.25%) were detected. That is why cellular phones are a potential source of microorganism causing nosocomial infections and the study indicates that proper hand hygiene and decontamination of devices should be carried out regularly.

A questionnaire was submitted to health care personnel and followed by the detection of bacterial growth in study [5]. There were questions about using headsets, disinfection of devices, hand hygiene, the location of using the devices. Fingers of both hands were examined for the presence of bacteria before and after phone call. It revealed that the bacterial growth was not present after hand rub, but after phone call the contamination increased to 93.7%.

Staphylococcus aureus was isolated form hands and mobile phones of health care workers according to [6]. *Methicillin Resistant Staphyococcus Aureus* (*MRSA*) was isolated from 53.3% mobile phones and it is unusual and reflects a high prevalence in the patient population of the hospital. Ethyl alcohol was effective as disinfecting agent.

The study [7] indicated that about 98% of cellular phones of health care workers were contaminated with bacteria, *ex Coagulase Negative Staphylococci*, *Staphylococcus aureus* , and *Escherichia coli*. The mobile phones were decontaminated with 70% alcohol, that decreased the rate of contamination from 98.3% to 55.2%.

In study [8] Of the mobile phones, 60% were contaminated, but drug resistant bacteria were not detected and 37% of individuals confirmed regular cleaning of devices.

A questionnaire asking about hygiene practice and the patters of using of mobile phones was used in [9]. A cross–sectional study was performed in the study and 61.7% of mobile phones showed contamination. It revealed that mobile phones of health care workers were more likely contaminated with *MRSA* versus devices of non–health care workers and generally the cellular phones of health care workers were more contaminated with bacteria

There was a study about the contamination of mobile phones in healthcare workers of neonatal unit [10]. The disinfection and cleaning reduces contamination of mobile devices and not using disinfection agents and not cleaning them cause the isolation of bacteria.

The study of comparison of flora associated with cellular phones in healthcare workers versus non–healthcare workers was described in [11]. Predominant microorganisms isolated from mobiles of healthcare workers were: *Acinetobacter baumannii* (36.84%) and *MRSA 46%* of samples from mobiles of non–healthcare workers harboured microorganisms.

Comparisons of microbial contamination of keypads and touch screens of mobile cell phones between hospital and non–hospital staffs were described in [12]. It was revealed that the microbial contamination of phones was low in touch screen devices and the bacterial contamination was more frequent in hospital staff devices. It was also observed that women's mobile phones were less contaminated than men's devices. In hospital staff mobile cell phones the dominant microorganism were: *Enterobacteriaceae , Bacillus species*, especially Gram–positive bacteria sporulated and *Coagulase Negative Staphylococcus*.

In the [13] investigation of microbial contamination of mobile phones in a tertiary care hospital results indicated that the growth on bacteria was present in 87.3% of mobile phones of heath care workers and 56.4% of the mobile phones of non–health care workers. *Coagulase Negative Staphylococcus* was predominant in the study group, aerobic spore bearers and micrococci were also frequent.

A cross sectional study was also performed in [14] 58% of health care workers' mobiles showed bacterial contamination with *Staphylococcus aureus* as a predominant bacterium (34%). Other detected bacteria were: *Micrococcus* species, *Coagulase Negative Staphylococcus* (*CNS*), *Pseudomonas aeruginosa* and *Escherichia coli*.

According to [15] 85% of the participants never cleaned their devices. Predominant bacterial isolates were *Staphylococcus aureus* and *Coagulase Negative Staphylococcus*, it was noted that 40% of *Staphylococcus aureus* isolates were resistant to methicillin. Gram negative bacteria are likely to represent poor hand hygiene.

The study [16] indicated that 81.8% of mobile phones and 80% of swab samples showed the growth of bacteria, where *Coagulase Negative Staphylococcus*, *Staphylococcus aureus*, *Acinetobacter* species, *Escherichia coli*, *Klebsiella pneumoniae*, *Pseudomonas* species and *Enterococcus* species were the most frequently isolated. That is why mobile phones may be a source of nosocomial pathogens.

2. Materials and Methods

The questions from the subset of the full survey considered in this paper are presented in the Table 1.

Table S 1: Questions considered in this paper.

**Question**	**Range**
(Q6) Smartphone market price	(300) $\cdots$ (>2100) PLN
(Q77) Did you lend your smartphone to another person?	never$\Longleftrightarrow$it happened often
(Q82) Do you ever use someone else's smartphone?	never$\Longleftrightarrow$it's not a problem
(Q83) Would you use someone else's smartphone that is dirty?	never$\Longleftrightarrow$it's not a problem
(Q84) Would you use someone else's smartphone that is wet with sweat?	never$\Longleftrightarrow$it's not a problem
(Q78) Would you lend your smartphone to a person who is visibly ~~sweaty~~sweating?	never$\Longleftrightarrow$it happened often
(Q79) Would you lend your smartphone to a visibly dirty person?	never$\Longleftrightarrow$it happened often
(Q80) Would you lend your smartphone to a person coughing or with a runny nose?	never$\Longleftrightarrow$it happened often
(Q81) Would you lend your smartphone to a person with visible skin changes?	never$\Longleftrightarrow$it happened often
(Q104 top) Do you think there are bacteria on touch screens?	none$\Longleftrightarrow$a lot of them
(Q104 middle) Do you think there are viruses on touch screens?	none$\Longleftrightarrow$a lot of them
(Q104 bottom) Do you think there are fungi on touch screens?	none$\Longleftrightarrow$a lot of them

PLN - Polish zloty (currency)

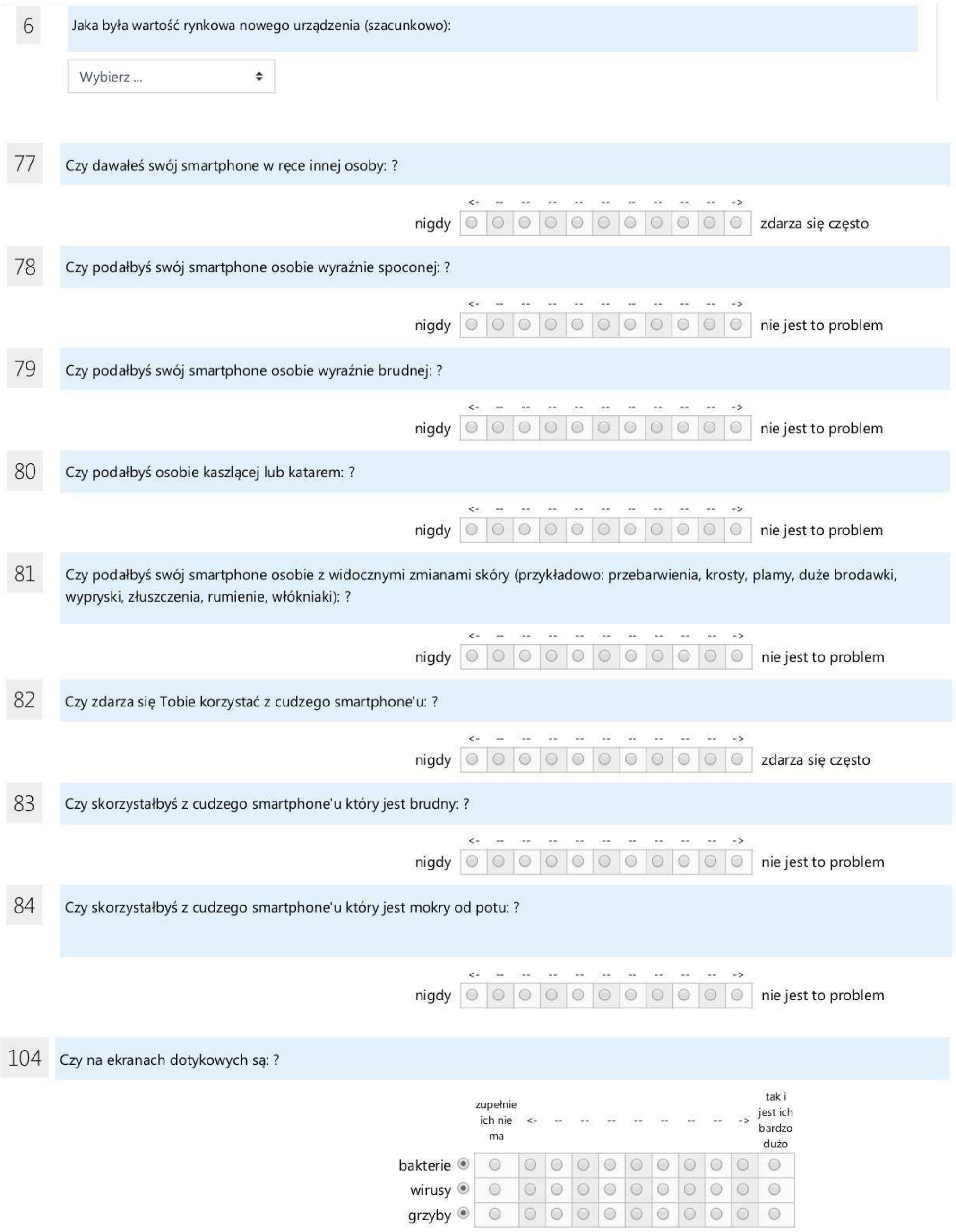

6 Jaka była wartość rynkowa nowego urządzenia (szacunkowo):

Wybierz ...

77 Czy dawałeś swój smartphone w ręce innej osoby: ?

nigdy <- -- -- -- -- -- -- -- -- -- -> zdarza się często

78 Czy podałbyś swój smartphone osobie wyraźnie spoconej: ?

nigdy <- -- -- -- -- -- -- -- -- -- -> nie jest to problem

79 Czy podałbyś swój smartphone osobie wyraźnie brudnej: ?

nigdy <- -- -- -- -- -- -- -- -- -- -> nie jest to problem

80 Czy podałbyś osobie kaszlącej lub katarem: ?

nigdy <- -- -- -- -- -- -- -- -- -- -> nie jest to problem

81 Czy podałbyś swój smartphone osobie z widocznymi zmianami skóry (przykładowo: przebarwienia, krosty, plamy, duże brodawki, wypryski, złuszczenia, rumienie, włókniaki): ?

nigdy <- -- -- -- -- -- -- -- -- -- -> nie jest to problem

82 Czy zdarza się Tobie korzystać z cudzego smartphone'u: ?

nigdy <- -- -- -- -- -- -- -- -- -- -> zdarza się często

83 Czy skorzystałbyś z cudzego smartphone'u który jest brudny: ?

nigdy <- -- -- -- -- -- -- -- -- -- -> nie jest to problem

84 Czy skorzystałbyś z cudzego smartphone'u który jest mokry od potu: ?

nigdy <- -- -- -- -- -- -- -- -- -- -> nie jest to problem

104 Czy na ekranach dotykowych są: ?

	zupełnie ich nie ma	<-	--	--	--	--	--	--	--	->	tak i jest ich bardzo dużo
bakterie											
wirusy											
grzyby											

Figure S1. Fragment of the questionnaire with the questions considered in the article (questionnaire in Polish).

3. Results

3.1. K–means and K–median Clustering of Social Behaviors Related to Giving Own Smartphone to Other Persons

Table S 2: Centroid locations for k–means.

**Number of clusters: k=3** centroid no.	value	**Number of clusters: k=4** centroid no.	value
**k–centroid type: k–means**			
Would you lend your smartphone to a person who is visibly sweating ? never (-1.0) ⟺ it happened often (1.0)			
suggested minus signs in all tables are ok [2]	-0.200	[1]	-0.418
		[2]	0.127
[3]	-0.725	[3]	-0.790
[1]	0.800	[4]	0.817
Would you lend your smartphone to a visibly dirty person ? never (-1.0) ⟺ it's not a problem (1.0)			
[2]	-0.482	[1]	-0.554
		[2]	-0.400
[3]	-0.832	[3]	-0.852
[1]	0.554	[4]	0.570
Would you lend your smartphone to a person coughing or with a runny nose ? never (-1.0) ⟺ it's not a problem (1.0)			
[2]	0.345	[1]	0.563
		[2]	-0.078
[3]	-0.668	[3]	-0.685
[1]	0.880	[4]	0.876
Would you lend your smartphone to a person with visible skin changes ? never (-1.0) ⟺ it's not a problem (1.0)			
[2]	-0.225	[1]	-0.009
		[2]	-0.618
[3]	-0.823	[3]	-0.819
[1]	0.760	[4]	0.776

Table S 3: Centroid locations for k–medians.

**Number of clusters: k=3**		**Number of clusters: k=4**	
centroid no.	value	centroid no.	value
**k–centroid type: k–medians**			
Would you lend your smartphone to a person who is visibly sweating ? never (-1.0) ⟺ it happened often (1.0)			
[2]	-0.2	[1]	-0.4
		[2]	0.0
[3]	-0.8	[3]	-0.8
[1]	1.0	[4]	1.0
Would you lend your smartphone to a visibly dirty person ? never (-1.0) ⟺ it's not a problem (1.0)			
[2]	-0.6	[1]	-0.4
		[2]	-0.6
[3]	-1.0	[3]	-1.0
[1]	0.8	[4]	1.0
Would you lend your smartphone to a person coughing or with a runny nose ? never (-1.0) ⟺ it's not a problem (1.0)			
[2]	0.4	[1]	0.6
		[2]	-0.2
[3]	-0.8	[3]	-0.8
[1]	1.0	[4]	1.0
Would you lend your smartphone to a person with visible skin changes ? never (-1.0) ⟺ it's not a problem (1.0)			
[2]	-0.4	[1]	0.0
		[2]	-0.8
[3]	-0.8	[3]	-1.0
[1]	1.0	[4]	1.0

3.2. Subjective Associations Between Expected Microbiological Threats and Behaviors using Linear Model

Table S 4: Absolute frequency for 2D linear model of associations (changed to italic$N$=172) (part 1/3)

y: Do you think there are bacteria/viruses/fungi on touch screens?			
		**Mean (SD)**	$Pr(>\|t\|)$
x: Smartphone market price PLN			
**bacteria**	Intercept:	-2.472638 (1.848149)	0.187
	x:	-0.000348 (0.000873)	0.692
	y:	8.951989 (1.948162)	3.26e-05 ***
**viruses**	Intercept:	1.073403 (0.566339)	0.0621 .
	x:	0.000298 (0.000335)	0.3770
	y:	1.657018 (0.594299)	0.0068 **
**fungi**	Intercept:	2.287e+00 (5.079e-01)	2.57e-05 ***
	x:	8.776e-05 (3.167e-04)	0.783
	y:	-1.523e-01 (5.490e-01)	0.782
x: Did you lend your smartphone to another person ? (never $\Longleftrightarrow$ it happened often)			
**bacteria**	Intercept:	-2.6421 (1.4627)	0.076774 .
	x:	1.6776 (0.7729)	0.034638 *
	y:	7.9599 (1.9375)	0.000145 ***
**viruses**	Intercept:	1.2649 (0.3245)	0.000202 ***
	x:	0.9856 (0.2799)	0.000717 ***
	y:	1.3994 (0.5195)	0.008619 **
**fungi**	Intercept:	2.0778 (0.2034)	< 2e-16 ***
	x:	0.7921 (0.1941)	0.000101 ***
	y:	-0.4274 (0.3575)	0.235210
x: Would you lend your smartphone to a person who is visibly sweating ? (never $\Longleftrightarrow$ it's not a problem)			
**bacteria**	Intercept:	-2.6437 (1.4397)	0.07203 .
	x:	-0.8927 (0.6741)	0.19120
	y:	7.6367 (1.8246)	0.00011 ***
**viruses**	Intercept:	2.0181 (0.4214)	9.06e-06 ***
	x:	-0.3315 (0.3078)	0.285
	y:	0.5000 (0.6368)	0.435
**fungi**	Intercept:	2.4472 (0.2894)	1.48e-12 ***
	x:	-0.5195 (0.2432)	0.0359 *
	y:	-0.6521 (0.4892)	0.1865

added:$Pr(>|t|)$ gives the p–value for that t–test (the proportion of the t distribution at that df which is greater than the absolute value of t statistic)
Signif. codes: '***' changed to italic$p \leq 0.001$, '**' $p \leq 0.01$, '*' $p \leq 0.05$, '.' $p \leq 0.1$, ' ' $p > 0.1$
PLN - Polish zloty (currency)

Table S 5: Absolute frequency for 2D linear model of associations (changed to italic*N*=172) (part 2/3).

y: Do you think there are bacteria/viruses/fungi on touch screens?			
		**Mean (SD)**	$Pr(>\|t\|)$
x: Would you lend your smartphone to a visibly dirty person ? (never $\Longleftrightarrow$ it's not a problem)			
**bacteria**	Intercept:	-3.9010 (1.9875)	0.05574 .
	x:	-2.3988 (0.9425)	0.01434 *
	y:	9.3391 (2.4976)	0.00051 ***
**viruses**	Intercept:	1.8567 (0.4465)	9.59e-05 ***
	x:	-0.9953 (0.3171)	0.00255 **
	y:	0.7462 (0.6748)	0.27292
**fungi**	Intercept:	2.5334 (0.3047)	3.97e-12 ***
	x:	-1.1753 (0.2581)	2.09e-05 ***
	y:	-0.9758 (0.5132)	0.0612 .
x: Would you lend your smartphone to a person coughing or with a runny nose ? (never $\Longleftrightarrow$ it's not a problem)			
**bacteria**	Intercept:	-2.5770 (1.4285)	0.076918 .
	x:	0.5885 (0.6831)	0.392861
	y:	7.9498 (1.9107)	0.000117 ***
**viruses**	Intercept:	1.4788 (0.3831)	0.000234 ***
	x:	0.2728 (0.2822)	0.336717
	y:	1.2200 (0.6126)	0.049956 *
**fungi**	Intercept:	2.3450 (0.2935)	8.45e-12 ***
	x:	0.3909 (0.2494)	0.121
	y:	-0.5530 (0.5133)	0.285
x: Would you lend your smartphone to a person with visible skin changes ? (never $\Longleftrightarrow$ it's not a problem)			
**bacteria**	Intercept:	-2.088 (1.448)	0.155272
	x:	-1.248 (0.650)	0.060178 .
	y:	7.001 (1.902)	0.000546 ***
**viruses**	Intercept:	1.3987 (0.3230)	4.34e-05 ***
	x:	-0.5588 (0.2502)	0.0284 *
	y:	1.1397 (0.5120)	0.0289 *
**fungi**	Intercept:	2.4107 (0.2716)	2.85e-13 ***
	x:	-0.8387 (0.2256)	0.000388 ***
	y:	-0.5339 (0.4790)	0.268606
x: Do you ever use someone else's smartphone ? (never $\Longleftrightarrow$ it's not a problem)			
**bacteria**	Intercept:	-2.3287 (1.0734)	0.0344 *
	x:	0.3559 (0.5154)	0.4927
	y:	7.4149 (1.4168)	2.68e-06 ***
**viruses**	Intercept:	1.5779 (0.2846)	3.87e-07 ***
	x:	0.3062 (0.2412)	0.2081
	y:	1.0620 (0.4482)	0.0203 *
**fungi**	Intercept:	2.08703 (0.20186)	<2e-16 ***
	x:	0.00136 (0.18260)	0.994
	y:	-0.37245 (0.35547)	0.298

added:$Pr(>|t|)$ gives the p–value for that t–test (the proportion of the t distribution at that df which is greater than the absolute value of t statistic)
Signif. codes: '***' changed to italic$p \leq 0.001$, '**' $p \leq 0.01$, '*' $p \leq 0.05$, '.' $p \leq 0.1$, ' ' $p > 0.1$

Table S 6: Absolute frequency for 2D linear model of associations (changed to italic$N$=172) (part 3/3).

y: Do you think there are bacteria/viruses/fungi on touch screens?			
		**Mean (SD)**	$Pr(>\|t\|)$
x: Would you use someone else's smartphone that is dirty ? (never $\Longleftrightarrow$ it's not a problem)			
**bacteria**	Intercept:	-2.0815 (1.1102)	0.0663 .
	x:	0.1105 (0.5294)	0.8355
	y:	7.1536 (1.4578)	9.2e-06 ***
**viruses**	Intercept:	1.4251 (0.2742)	1.51e-06 ***
	x:	0.1981 (0.2177)	0.36566
	y:	1.2240 (0.4399)	0.00673 **
**fungi**	Intercept:	2.09286 (0.20382)	2.91e-16 ***
	x:	0.34832 (0.18051)	0.0572 .
	y:	0.01384 (0.37325)	0.9705
x: Would you use someone else's smartphone that is wet with sweat ? (never $\Longleftrightarrow$ it's not a problem)			
**bacteria**	Intercept:	-4.1361 (1.3420)	0.003312 **
	x:	-2.2725 (0.6409)	0.000849 ***
	y:	9.6104 (1.7407)	1.14e-06 ***
**viruses**	Intercept:	1.5417 (0.3921)	0.000196 ***
	x:	-0.9295 (0.3001)	0.002810 **
	y:	1.0380 (0.6041)	0.090133 .
**fungi**	Intercept:	2.2300 (0.2427)	5.24e-14 ***
	x:	-1.0951 (0.2241)	5.47e-06 ***
	y:	-0.6505 (0.4077)	0.115

added:$Pr(>|t|)$ gives the p–value for that t–test (the proportion of the t distribution at that df which is greater than the absolute value of t statistic)
Signif. codes: '***' changed to italic$p \leq 0.001$, '**' $p \leq 0.01$, '*' $p \leq 0.05$, '.' $p \leq 0.1$, ' ' $p > 0.1$

91 *3.3. Subjective Associations between Microbiological Threats for Personal Contact Situations*
92 *using Linear Model*

Table S 7: Absolute frequency for 2D linear model of associations (changed to italic$N$=172).

x:	Did you lend your smartphone to another person ?	
	**Mean (SD)**	$Pr(>\|t\|)$
y: Would you lend your smartphone to a person who is visibly sweating ? (never $\Longleftrightarrow$ it's not a problem)		
Intercept:	2.4154 (0.2837)	3.57e-12 ***
x:	0.4029 (0.4455)	0.369
y:	-0.2379 (0.4070)	0.561
y: Would you lend your smartphone to a visibly dirty person ? (never $\Longleftrightarrow$ it's not a problem)		
Intercept:	2.4044 (0.2938)	3.4e-11 ***
x:	0.8876 (0.4437)	0.0502 .
y:	-1.1045 (0.4211)	0.0112 *
y: Would you lend your smartphone to a person coughing or with a runny nose ? (never $\Longleftrightarrow$ it's not a problem)		
Intercept:	2.3818 (0.3137)	1.3e-10 ***
x:	0.7232 (0.5391)	0.184
y:	0.5567 (0.4580)	0.228
y: Would you lend your smartphone to a person with visible skin changes ? (never $\Longleftrightarrow$ it's not a problem)		
Intercept:	2.2120 (0.2247)	8.95e-15 ***
x:	1.0402 (0.3694)	0.00634 **
y:	-0.7552 (0.3245)	0.02288 *
y: Do you ever use someone else's smartphone ? (never $\Longleftrightarrow$ it's not a problem)		
Intercept:	2.3785 (0.2644)	3.04e-13 ***
x:	0.5773 (0.4928)	0.245
y:	0.5333 (0.4735)	0.264
y: Would you use someone else's smartphone that is dirty ? (never $\Longleftrightarrow$ it's not a problem)		
Intercept:	2.0940 (0.2368)	8.32e-13 ***
x:	1.0398 (0.3655)	0.00591 **
y:	-1.1551 (0.3549)	0.00179 **

Signif. codes: '***' changed to italic$p \leq 0.001$, '**' $p \leq 0.01$, '*' $p \leq 0.05$, '.' $p \leq 0.1$, ' ' $p > 0.1$

References

1. Szabó, R.; Morvai, J. The role of the mobile communication devices in the spread of healthcare-associated infections: a systematic review. *Antimicrobial Resistance and Infection Control* **2015**, *4*, P39.
2. Goldblatt, J.G.; Krief, I.; Klonsky, T.; Haller, D.; Milloul, V.; Sixsmith, D.M.; Srugo, I.; Potasman, I. Use of Cellular Telephones and Transmission of Pathogens by Medical Staff in New York and Israel. *Infection Control & Hospital Epidemiology* **2007**, *28*, 500–503.
3. Usha, A.; Pushpa, D.; Aarti, C.; Sita, M. Cellphones A Modern Stayhouse For Bacterial Pathogens. *JK Science : Journal of Medical Education & Research* **2009**, *11*, 127–129.
4. Kokate, S.; More, S.; Gujar, V.; Mundhe, S.; Quazi Syed, Z. Microbiological flora of mobile phones of resident doctors. *J. Biomedical Science and Engineering* **2012**, *5*, 696–698.
5. Badr, R.; Badr, H.; Mansour, N. Mobile phones and nosocomial infections. *International Journal of Infection Control* **2012**, *8*, 1–5.

6. Angadi, K.; Gupta, U.; Misra, R.; Jadhav, S.; Sardar, M. Study of the role of mobile phones in the transmission of Hospital acquired infections. *Medical Journal of Dr. D.Y. Patil University* **2014**, *7*, 435–438.
7. Gashaw, M.; Abtew, D.; Addis, Z. Prevalence and Antimicrobial Susceptibility Pattern of Bacteria Isolated from Mobile Phones of Health Care Professionals Working in Gondar Town Health Centers. *ISRN Public Health* **2014**, *2014*, 1–6.
8. Mark, D.; Leonard, C.; Breen, H.; Graydon, R.; O'Gorman, C.; Kirk, S. Mobile phones in clinical practice: reducing the risk of bacterial contamination. *International journal of clinical practice* **2014**, *68*, 1060–1064.
9. Misgana, G.; Abdissa, K.; Abebe, G. Bacterial contamination of mobile phones of health care workers at Jimma University Specialized Hospital, Jimma, South West Ethiopia. *International Journal of Infection Control* **2015**, *11*.
10. Orsi, G.; Natale, F.; D'Ettorre, G.; Protano, C.; Vullo, V.; De Curtis, M. Mobile Phone Microbial Contamination Among Neonatal Unit Healthcare Workers. *Infection Control and Hospital Epidemiology* **2015**, *36*, 487–489.
11. Morubagal, R.R.; Shivappa, S.G.; Mahale, R.P.; Neelambike, S.M. Study of bacterial flora associated with mobile phones of healthcare workers and non–healthcare workers. *Iranian journal of microbiology* **2017**, *9*, 143–151.
12. Dorost, A.; Safari, Y.; Akhlaghi, M.; Soleimani, M.; Yoosefpour, N. Microbial contamination data of keypad and touch screen of cell phones among hospital and non-hospital staffs - A case study: Iran. *Data in brief* **2018**, *20*, 80–84.
13. Anupriya, A.; Puhalenthi, K.; S., J.K.; R., P.; V., H. Microbial contamination of mobile phones in a teritary care hospital. *International Journal Of Community Medicine And Public Health* **2018**, *5*, 2313–2316.
14. Ebidor, U. Mobile phones of health care workers: Friend or foe. *Global journal of Research Analysis* **2015**, *4*, 1–2.
15. Chaka, T.; Milisa, G.; Feye, B.; Kassa, R. Bacterial Isolates from Cell Phones and Hands of Health Care Workers: A Cross Sectional Study in Pediatric Wards at Black Lion Hospital, Addis Ababa, Ethiopia. *Journal of Bacteriology & Parasitology* **2016**, *7*, 288–294.
16. Pal, S.; Juyal, D.; Adekhandi, S.; Sharma, M.; Prakash, R.; Sharma, N.; Rana, A.; Parihar, A. Mobile phones: Reservoirs for the transmission of nosocomial pathogens. *Advanced biomedical research* **2015**, *4*, 144–144.
