# Supplementary material for: The Associations between Knowledge and Behaviours Related to Touch Screens and Microbiological Threats among IT Students’
Source: Int J Environ Res Public Health. 2021 Sep 2;18(17):9269. doi: 10.3390/ijerph18179269 (PMC8431698; doi:10.3390/ijerph18179269)
Supplement: Supplementary file 1 [file ijerph-18-09269-s001.zip › questions.pdf]

6 Jaka była wartość rynkowa nowego urządzenia (szacunkowo):

Wybierz ...

77 Czy dawałeś swój smartphone w ręce innej osoby: ?

<- -- -- -- -- -- -- -- -- -- ->
nigdy ○ ○ ○ ○ ○ ○ ○ ○ ○ ○ ○ zdarza się często

78 Czy podałbyś swój smartphone osobie wyraźnie spoconej: ?

<- -- -- -- -- -- -- -- -- -- ->
nigdy ○ ○ ○ ○ ○ ○ ○ ○ ○ ○ ○ nie jest to problem

79 Czy podałbyś swój smartphone osobie wyraźnie brudnej: ?

<- -- -- -- -- -- -- -- -- -- ->
nigdy ○ ○ ○ ○ ○ ○ ○ ○ ○ ○ ○ nie jest to problem

80 Czy podałbyś osobie kaszlącej lub katarem: ?

<- -- -- -- -- -- -- -- -- -- ->
nigdy ○ ○ ○ ○ ○ ○ ○ ○ ○ ○ ○ nie jest to problem

81 Czy podałbyś swój smartphone osobie z widocznymi zmianami skóry (przykładowo: przebarwienia, krosty, plamy, duże brodawki, wypryski, złuszczenia, rumienie, włókniaki): ?

<- -- -- -- -- -- -- -- -- -- ->
nigdy ○ ○ ○ ○ ○ ○ ○ ○ ○ ○ ○ nie jest to problem

82 Czy zdarza się Tobie korzystać z cudzego smartphone'u: ?

<- -- -- -- -- -- -- -- -- -- ->
nigdy ○ ○ ○ ○ ○ ○ ○ ○ ○ ○ ○ zdarza się często

83 Czy skorzystałbyś z cudzego smartphone'u który jest brudny: ?

<- -- -- -- -- -- -- -- -- -- ->
nigdy ○ ○ ○ ○ ○ ○ ○ ○ ○ ○ ○ nie jest to problem

84 Czy skorzystałbyś z cudzego smartphone'u który jest mokry od potu: ?

<- -- -- -- -- -- -- -- -- -- ->
nigdy ○ ○ ○ ○ ○ ○ ○ ○ ○ ○ ○ nie jest to problem

104 Czy na ekranach dotykowych są: ?

		zupełnie ich nie ma	<-	--	--	--	--	--	--	--	->	tak i jest ich bardzo dużo
bakterie	◉	○	○	○	○	○	○	○	○	○	○	○
wirusy	◉	○	○	○	○	○	○	○	○	○	○	○
grzyby	◉	○	○	○	○	○	○	○	○	○	○	○
