# Supplementary figures and images for: The Associations between Knowledge and Behaviours Related to Touch Screens and Microbiological Threats among IT Students’
Source: Int J Environ Res Public Health. 2021 Sep 2;18(17):9269. doi: 10.3390/ijerph18179269 (PMC8431698; doi:10.3390/ijerph18179269)

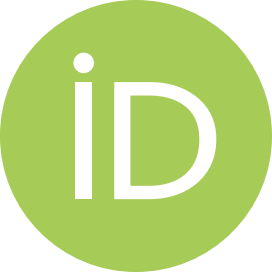

Supplement: Supplementary file 1 [file ijerph-18-09269-s001.zip › Definitions/logo-orcid-eps-converted-to.pdf]

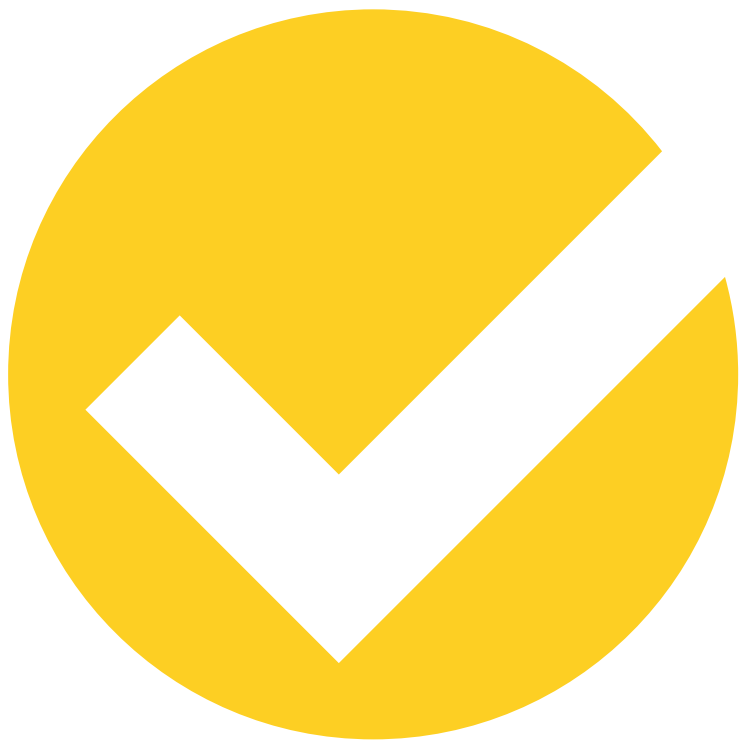

check for  
updates

Supplement: Supplementary file 1 [file ijerph-18-09269-s001.zip › Definitions/logo-updates.pdf]
